# Implications of neuropathy and management of the corneal surface in a patient with Stuve-Wiedemann syndrome

**DOI:** 10.22336/rjo.2023.66

**Published:** 2023

**Authors:** María Larrañaga Cores, Ana Boto de los Bueis

**Affiliations:** * Ophthalmology Service, Hospital Universitario La Paz, Madrid, Spain

**Keywords:** Stuve Wiedemann, persistent epithelial defects, exposure keratopathy, confocal microscopy, neuropathy

## Abstract

**Purpose:** To describe the ophthalmological management of a girl diagnosed with Stuve Wiedemann syndrome (SWS). Clinical and in vivo confocal microscopy (IVCM) are described.

**Methods:** Case report of a 6-year-old girl, who presented with neurotrophic keratitis and was treated with intense lubrication including heterologous serum and tear plugs.

**Results:** In the following months, the evolution of the neurotrophic keratitis was good, but a hypertrophic corneal leukoma persisted with mild neovascularization in the left eye.

**Conclusion:** Close ophthalmological follow-up in patients with SWS is needed, given that most of the time they do not present symptoms due to the characteristic neuropathy of their lesions.

**Abbreviations:** SWS = Stuve-Wiedemann syndrome, IVCM = in vivo confocal microscopy, CNTF = ciliary neurotrophic factor, BCVA = best corrected visual acuity, LIFR = Leukemia Inhibitory Factor Receptor, IGF1 = Insulin-like growth factor-1

## Introduction

Stuve-Wiedemann syndrome (SWS) is a rare and severe autosomal recessive disease. It is characterized by skeletal anomalies such as bowed long bones or joint restrictions; and dysautonomia disturbances with ocular and neuropathic features due to disorders in the ciliary neurotrophic factor (CNTF) receptor pathway [**1**-**3**]. It leads to respiratory and feeding difficulties and hyperthermia episodes, usually causing death in the first two years of life [**2**-**4**]; although a series of long-term survivors have been reported [**1**].

In the ophthalmological field, the scarce tear production, corneal anesthesia, and the absence of the blink reflex due to dysautonomia cause various corneal alterations that in the most severe cases can lead to the presence of neurotrophic ulcers or important corneal opacities [**1**,**2**,**5**]. Management of these problems from the ophthalmology consultation is important both for the patient’s quality of life and to avoid problems such as ocular perforation [**1**,**3**]. 

We present a case of a 6-year-old girl with SWS with keratitis and bilateral corneal scarring secondary to the alteration of corneal reflexes, associated with reduced tear secretion, who has been under treatment and follow-up for 2 years by the cornea section of our service. We describe the therapeutic steps followed until the presentation of our case report and their results.

## Case report

A 6-year-old girl diagnosed with Stuve-Wiedemann Syndrome was referred for ophthalmological evaluation during a scheduled admission after trauma surgery.

At the initial presentation, we measured the best corrected visual acuity (BCVA) for the right eye: 0.7 and the left eye: 0.5. Refraction under cycloplegia was as follows: in her right eye: +2.25, -3.75 x 178º, and in her left eye +0.50, -4.50 x 180º. On examination with a slit lamp, we found that the cornea in the right eye presented a mild-density opacity lower paracentral, horizontally arranged and with an extension of 1 mm; and in the left eye a diffuse mild opacity, occupying the entire length of the cornea. The rest of the ophthalmological examination was unremarkable. With the diagnosis of bilateral mild corneal opacities and astigmatism associated with Stuve-Wiedemann Syndrome, we prescribed treatment with artificial tears.

**Fig. 1:**
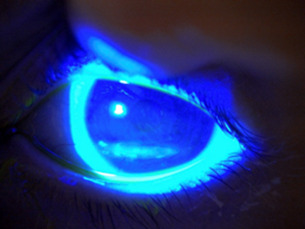
Hypertrophic corneal leukoma in the lower third of the left eye

Four months later, the patient’s BCVA decreased to 0.5 in the right eye and 0.125 in the left eye. On examination, the patient presented diffuse superficial punctate keratitis grade IV on the Oxford scale in both eyes. Worsening of the inferior leukoma in both eyes was observed, especially in her left eye: objectifying a hypertrophic inferior leukoma, probably due to nocturnal exposure (**Fig. 1**). An incomplete blinking was also observed, which combined with the absence of pain or ocular discomfort, suggested probable neurotrophy. The Schirmer test was altered on her left eye (right eye 11 mm and left eye 6 mm) and no alteration of the meibomian glands was observed, which suggested insufficient tear production. We decided to treat our patient with abundant preservative-free artificial tears with hyaluronic acid during the day and ointment with ocular occlusion at night. In addition, 3,35 mg/ml hydrocortisone eye drops were added in a descending pattern starting with an application every 8 hours in both eyes.

We reviewed the patient 3 months later, observing an improvement in the keratitis with persistence of the inferior leukoma in both eyes. Keratometry was as follows:

- Right eye: 42.25 x 45.25 at 87;

- Left eye: 39.25 x 42.25 at 96.

**Fig. 2:**
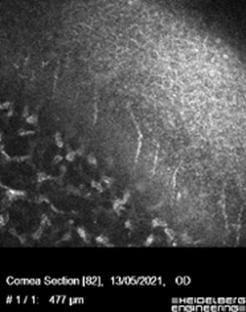
IVCM image showing the presence of a sub-basal nerve plexus with decreased density

**Fig. 3:**
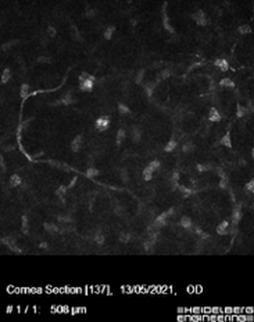
IVCM image showing one nerve in the anterior stroma

In vivo confocal microscopy (IVCM) was performed, showing a decreased density in the branches of the sub-basal nerve plexus. The size and morphology were altered in the superficial, intermediate, and basal epithelial cells (**Fig. 2**). Isolated nerves were detected at the anterior stroma (**Fig. 3**).

Treatment with artificial tears and night ointment was maintained, and the placement of tear plugs was associated. Treatment with heterologous serum from the mother was also included.

The evolution of the neurotrophic keratitis in the following months was good, with a notable improvement in the keratitis, but a hypertrophic linear corneal leukoma persisted in the lower third with mild neovascularization in the left eye. Her BCVA in the last ophthalmological examination was 0.4 in the right eye and 0.5 in the left eye. However, given the neurotrophic keratitis presented, the persistence of corneal irregularity, and the mild bilateral keratitis with corneal opacities, we decided to start treatment with insulin eye drops, added to the treatment with artificial tears, heterologous serum, and night ointment; treatment with which the patient was controlled without any adverse effects until the moment of the case presentation.

## Discussion

While the natural history of SWS is marked by a high mortality rate in the first two years of life [**1**,**2**,**4**], the case we report describes a 6-year-old patient diagnosed with SWS. In the literature, only a few cases describe survivors older than 16 years of age [**5**]. The main causes of death at an early age are respiratory failure or difficulties with feeding and swallowing in the neonatal period [**1**,**2**], and, after this period, episodes of hyperthermia due to failures in the regulation of body temperature as a symptom of dysautonomia [**1**,**2**]. Traumatological symptoms become important after two years of age, as well as ophthalmological ones: both being the main concern in our patients nowadays [**1**]. 

SWS is considered a rare disorder, whose prevalence is unknown [**2**]. Regarding the genetic basis of this syndrome, the mutation of both copies of the Leukemia Inhibitory Factor Receptor (LIFR) gene, located on chromosome 5p13, has been described as the causal agent [**1**,**2**,**6**]. It is caused by a “null” mutation that results in a lack of function for the LIFR gene [**6**]. LIFR binds multiple cytokines in the human body, thus its absence causes the characteristic problems of SWS [**2**]. Specifically, autonomic nerve dysfunction is thought to be caused by non-adherence of LIFR to CNTF, although more studies are necessary [**2**]. The high prevalence of consanguinity in patients diagnosed with SWS is consistent with the autosomal recessive inheritance that it presents [**1**,**2**], although genetic and phenotypic heterogeneity has been described in this syndrome [**1**]. Discussion of the specific risk that involves the use of general anesthesia in this patient has been evaluated. Since one of the main symptoms of SWS is hyperthermia, it was hypothesized that there might be an association between SWS and malignant hyperthermia [**2**]. However, Bonthuis D et al. studied a possible genetic association between SWS and malignant hyperthermia, concluding that the molecular and genetic mechanism is different: malignant hyperthermia is a pharmacological disorder associated with autosomal dominant inheritance of mutations in the RYR1 receptor and the CACNA1S gene [**6**]. Therefore, there would be no strict contraindication for the use of inhaled anesthetics in surgery in patients with SWS [**6**]. 

As there is no specific treatment for this disease, medical action aims to mitigate the symptoms of the disease and to avoid complications [**2**]. 

In the field of ophthalmology, the main manifestations of dysautonomia described are corneal ulcers and opacities due to alacrimia or hypolacrimation and corneal anesthesia that lead to a decrease in the blink reflex [**1**-**3**,**5**]. Conjunctival staining or complete lid closure during sleep has also been described [**3**]. Since these complications occur at an early age, we must pay close attention to these irregularities and corneal opacities, as they are likely to cause amblyopia [**1**-**3**]. Treatment depends on the stage of keratitis and the dysautonomia symptoms present in each patient [**7**] and includes artificial drops and ointments or surgical procedures if necessary (punctual occlusion, lateral tarsorrhaphy or optical iridectomy if the corneal opacity covers the visual axis) [**1**,**2**]. 

The cornea is the most innervated tissue in the human body, and when neurotrophic keratitis occurs because of corneal anesthesia the corneal epithelium is the first affected, showing dystrophic changes that do not heal spontaneously and may lead to corneal ulcers, melting, and perforation if left untreated [**7**]. 

Mackie’s classification of neurotrophic keratitis is useful for establishing the different types of treatment for each stage [**7**]. According to this classification, stage 1 describes changes such as epithelial punctate keratitis, epithelial hyperplasia, irregularity, or neovascularization with stromal scarring; stage 2 corresponds to a persistent epithelial defect and stage 3 includes stromal involvement in an ulcer with melting or risk of perforation [**7**].

According to this classification, the first therapeutic step in SWS corneal involvement usually includes conservative procedures aimed at improving the lubrication of the corneal surface, such as preservative-free artificial tears and night ointment [**2**,**3**,**7**], as we prescribed at the first moment in our patient. Silicone punctal plugs can be used to improve lubrication [**3**].

When ulcers that do not heal with this conservative management occur, it may be necessary to perform surgical procedures, among which tarsorrhaphy, coverage of the defect with an amniotic membrane transplant, or injection of botulinum toxin in the eyelid elevator to produce a ptosis that keeps the eye occluded have been described as useful [**2**,**3**,**7**]. Nevertheless, we must make a correct balance of benefit and risk since with that type of procedure the visual function is limited in children because of the risk of amblyopia. Corneal neurotization surgery has not been described in this syndrome, but theoretically, its results could be compromised due to the diffuse neural affectation of the disease. Variable success has been described with this treatment in a few children diagnosed with different congenital diseases [**1**,**8**]. 

Low-dose topical corticosteroids have been used to reduce epithelial scarring, as they inhibit inflammatory mediators such as prostaglandins that retard epithelial growth [**3**,**7**]. In recent years, successful results have been published in the re-epithelialization of neurotrophic corneal ulcers with the use of topical insulin, although more studies on its efficacy and side effects are still necessary [**9**]. The mechanism is not fully understood yet, but it is hypothesized that insulin helps restore corneal nerves and promotes epithelial cell migration [**9**]. Some studies suggest that Insulin-like growth factor-1 (IGF1) treatment may also be beneficial in corneal epithelium healing in neurotrophic keratitis [**7**,**9**]. 

In these patients, surgical procedures aimed at restoring corneal transparency like any type of corneal transplant should be avoided given the recurrent nature of the pathology and due to the high risk of epithelial defect, ulcers, corneal melting, and perforation after surgery [**5**,**7**]. 

We must not forget that the second part of the treatment in children with SWS is to help the development of vision, allowing the greatest amount of light to stimulate the retina [**3**]. For this, treatment with 1% tropicamide eye drops or even iridectomy in cases of central corneal opacities may be useful [**3**]. 

## Conclusion

Our case highlighted the importance of a close ophthalmological follow-up in patients with SWS, given that most of the time they do not present symptoms due to the characteristic neuropathy of their lesions, and because they are usually children who do not manifest their discomfort. It is important to check and prescribe a correct refraction in each of the visits, to avoid amblyopia in these children. The first therapeutic step should include preservative-free artificial tears and night ointment, and mild corticosteroids may be associated for a short period to break the inflammatory cycle and reduce corneal scarring. Surgical procedures such as tarsorrhaphy or iridectomy may be necessary in more advanced stages. Corneal transplant is contraindicated given the nature of the disease, which would lead to postoperative failure.

Although episodes of hyperthermia in SWS have not been related to general anesthesia, surgery in these patients may be compromised given the poor corneal healing characteristic of the disease and its dysautonomia.

**Conflict of Interest Statement**

The authors state no conflict of interest. 

**Informed Consent and Human and Animal Rights Statement**

An informed consent has been obtained from the patient and family.

**Authorization for the use of human subjects**

Ethical approval: The research related to human use complies with all the relevant national regulations and institutional policies, as per the tenets of the Helsinki Declaration, and has been approved by the Ethics Committee of La Paz University Hospital, Madrid, Spain.

**Acknowledgments**

None.

**Sources of Funding**

None.

**Disclosures**

None.
